# Analgesic effects of transcutaneous auricular vagus nerve stimulation on partial sciatic nerve ligation-induced neuropathic pain in mice via serotonergic pathways

**DOI:** 10.1186/s13041-025-01246-2

**Published:** 2025-09-26

**Authors:** Hyunjin Shin, Seunghwan Choi, Geehoon Chung, Sun Kwang Kim

**Affiliations:** 1 Department of Science in Korean Medicine, Graduate School, Kyung Hee University, Seoul, 02447 Korea; 2 Department of Physiology, College of Korean Medicine, Kyung Hee University, Seoul, 02447 Korea; 3 Neurogrin Inc., Seoul, 02447 Korea; 4 Department of Physiology, Chungbuk National University College of Medicine, Cheongju, 28644 Korea

**Keywords:** Transcutaneous auricular vagus nerve stimulation (taVNS), Partial sciatic nerve ligation (PSL), Mechanical allodynia, Serotonin pathway, Dorsal raphe nucleus (DRN), Central amygdala (CeA)

## Abstract

Current treatments for neuropathic pain often provide limited relief and are associated with significant side effects. Transcutaneous auricular vagus nerve stimulation (taVNS) shows promise as a non-pharmacological analgesic approach; however, its optimal therapeutic configuration and underlying brain mechanisms remain incompletely understood. This study investigated the analgesic effects of taVNS on neuropathic pain in a mouse model induced by partial sciatic nerve ligation (PSL), exploring mechanisms and optimizing configurations. PSL-induced neuropathic pain in mice, characterized by mechanical allodynia, was significantly alleviated by taVNS. The most robust analgesic effects were observed with multiple bilateral taVNS sessions, administered once daily for three consecutive days, with effects persisting for at least 48 h post-stimulation. Immunohistochemical analysis of c-Fos expression revealed that taVNS increased neural activity in the dorsal raphe nucleus (DRN), a key source of serotonin, while simultaneously reducing activity in the central amygdala (CeA), a region critical for pain processing and affective responses. Further experiments demonstrated that the analgesic effects of taVNS were abolished by systemic administration of p-chlorophenylalanine, an inhibitor of serotonin synthesis. These findings underscore the critical role of serotonin signaling in mediating taVNS-induced analgesia for neuropathic pain. The study also highlights the importance of stimulation parameters, identifying a multiple bilateral configuration as particularly effective. Our results suggest that taVNS, potentially acting via the DRN-serotonergic system to modulate limbic structures like the CeA, holds significant potential as a non-pharmacological therapeutic option for managing neuropathic pain.

## Introduction

Chronic neuropathic pain, a pathological condition caused by damage or disease of the somatosensory nervous system, has long been recognized as a significant medical issue [1]. It manifests symptoms including spontaneous burning pain and hypersensitivity to external stimuli (e.g. various forms of hyperalgesia and allodynia). Globally, an estimated 7–10% of the population experiences neuropathic pain, with 20–30% of these cases progressing to chronic pain [1, 2]. The causes of neuropathic pain are numerous and varied [3], and patients often suffer from intractable pain and accompanying other symptoms including sleep disturbances, anxiety, and depression. Consequently, their quality of life is more severely impaired than that of patients with other types of pain [2].

Despite current pharmacological interventions, treatments often fail to provide adequate or lasting pain relief [4–6]. Gabapentin and pregabalin, initially developed as anticonvulsants, are widely prescribed for neuropathic pain. In addition, antidepressants like tricyclic antidepressants, selective serotonin reuptake inhibitors, and serotonin-norepinephrine reuptake inhibitors are frequently used. However, their efficacy is limited to a subset of patients, and they often cause adverse effects including drowsiness, dizziness, nausea, vomiting, and indigestion; severe cases can involve angioedema and cardiovascular complications. Opioid use is limited due to the risk of addiction and respiratory depression, which can be fatal in cases of overdose [7–9]. Furthermore, inappropriate dosing of recommended drugs contributes to poor therapeutic outcomes in some patients [4]. Given these limitations, neuromodulation techniques that do not rely on pharmacological interventions, such as vagus nerve stimulation (VNS), are emerging as promising alternatives.

VNS has demonstrated efficacy in treating epilepsy, depression, and improving cognitive function [10, 11]. VNS has also shown promise in alleviating pain in specific neuropathic conditions, such as chemotherapy-induced peripheral neuropathy (CIPN) [12, 13] and diabetic neuropathy [14]. Notably, transcutaneous auricular vagus nerve stimulation (taVNS) is a non-invasive VNS method stimulating the auricular branch of the vagus nerve, offering a safer and more convenient alternative to surgical cervical VNS.

The neuronal activation signal generated by taVNS passes through the nucleus tractus solitarius (NTS) in the medulla, then projects to the locus coeruleus (LC) and the dorsal raphe nucleus (DRN), subsequently spreading to various brain regions [15]. This study aimed to investigate the analgesic effects of taVNS on neuropathic pain, focusing on the associated brain pathways and neurotransmitters. Specifically, we examined the roles of the DRN, a key region for the serotonergic pain modulation [16], and the central amygdala (CeA), a region critical for emotion and pain perception.

## Materials and methods

### Animals

Male C57BL/6 mice aged 5–6 weeks were used for the studies. These mice were acquired from Koatech (Pyeongtaek, Korea) and were housed four per cage to minimize stress. They were maintained on a 12-h light/dark cycle and kept at 23 ± 2 °C. All experiments were conducted during daylight hours to reduce the effects of diurnal variation. All animal procedures were carried out in accordance with the protocols approved by the Institutional Animal Care and Use Committee of Kyung Hee University (KHUASP-24-024). The mice were given a 1-week to acclimate period before beginning the experiments. For all experiments, animals were randomly assigned to a group, and the experimenter was blinded to the group assignment.

### PSL surgery

Before surgery, mice were anesthetized using 2% isoflurane with O_2_ and N_2_O. To induce neuropathic pain through nerve injury, the right sciatic nerve was ligated with a 7–0 suture at approximately one-third to one-half of its diameter after incising the upper thigh. In the sham group, the same procedure was performed without manipulating the nerve [17].

### Mechanical allodynia behavior test

Before evaluating mechanical allodynia, the mice were acclimated to the metal mesh chamber. They underwent an acclimatization period in which they were placed in an opaque plastic box (12 × 8 × 6 cm) for 2 h per day over the course of 3 days. Mechanical allodynia induced by PSL was evaluated by applying von Frey filaments (bending force to 2.36, 2.44, 2.83, 3.22, 3.61, 3.84, 4.08, and 4.31 expressed as the log of the bending force in gram) to the mid-plantar surface of the right hind paw. The assessment applied the up-down method to determine the threshold force corresponding to a 50% withdrawal response [18, 19]. The experimenters were blinded to the treatments administered to the animals.

### TaVNS

Mice were anesthetized under 2% isoflurane with O_2_ and N_2_O. Electrical stimulation was delivered to the cymba concha, an area innervated by the auricular branch of the vagus nerve [20], using a USB Multifunction 2-channel Arbitrary Waveform Generator (Digistim, npi electronic GmbH, Germany). Magnetic electrodes were bilaterally attached using alligator clips, and stimulation was applied for 20 min [10]. The stimulation parameters were set to 5 volts, with a square waveform at 20 Hz. The control group underwent the same procedure without electrical stimulation.

### Serotonin depletion

To investigate the involvement of serotonin in the effect of taVNS, 4-Chloro-DL-phenylalanine methyl ester hydrochloride (PCPA; Sigma, St. Louis, MO, USA), a serotonin synthesis inhibitor, was intraperitoneally administered to the partial sciatic nerve ligation model. PCPA was dissolved in phosphate-buffered saline (PBS) [21]. To assess serotonin depletion on the taVNS effect, PCPA (30 mg/kg) or 1x PBS was administered 30 min before the taVNS treatment in PSL group for 3 days.

### Immunohistochemistry (IHC)

To identify brain regions involved in the analgesic effects induced by taVNS, immunohistochemistry was conducted 2 h after the stimulation session. Mice were deeply anesthetized with isoflurane and perfused transcardially with ice-cold phosphate buffered saline (PBS), followed by 4% paraformaldehyde.

Brains were extracted and stored in 4% paraformaldehyde overnight for fixation. The following day, brains were transferred to a 30% sucrose solution for dehydration and stored at 4 °C until they sank. The fixed brain was embedded in an OCT mounting medium and sectioned using a cryostat at a thickness of 30 μm. After several washes with PBS, the sections were incubated with a solution containing 0.5% bovine serum albumin (BSA) and 0.3% Triton X-100 in PBS. To confirm neuronal localization of c-Fos, the sections were treated with primary antibodies (rabbit anti-NeuN, 1:1000, 702022, Thermo; rat anti-c-Fos, 1:1000, 226 017, Synaptic Systems), conjugated with secondary antibodies (Alexa 546 and Alexa 488, 1:500, Invitrogen). All sections were mounted with a mounting medium containing DAPI (H-1200-10, Vector Laboratories, Inc., USA). The slides were scanned using a confocal laser scanning microscopy system (LSM800 with Airyscan, Carl Zeiss Microscopy, Oberkochen, Germany) [22].

### Image analysis

Fluorescent images were analyzed using QuPath v0.5.1 [23] after defining regions of interest (ROIs) based on the mouse brain atlas. The *Positive Cell Detection* function in QuPath was used to automatically count NeuN-positive and c-Fos-positive cells within each ROI. The density of c-Fos-positive cells was calculated as the percentage of NeuN-positive cells (c-Fos⁺/NeuN⁺). Statistical analyses were performed using GraphPad Prism 10.2.3 (GraphPad Software, Inc., USA). Comparisons between the PSL and PSL + taVNS groups were made using an unpaired t-test. Data are presented as mean ± standard error of the mean (SEM), and a p-value < 0.05 was considered statistically significant.

## Results

### Induction of chronic neuropathic pain via PSL

**Fig. 1:**
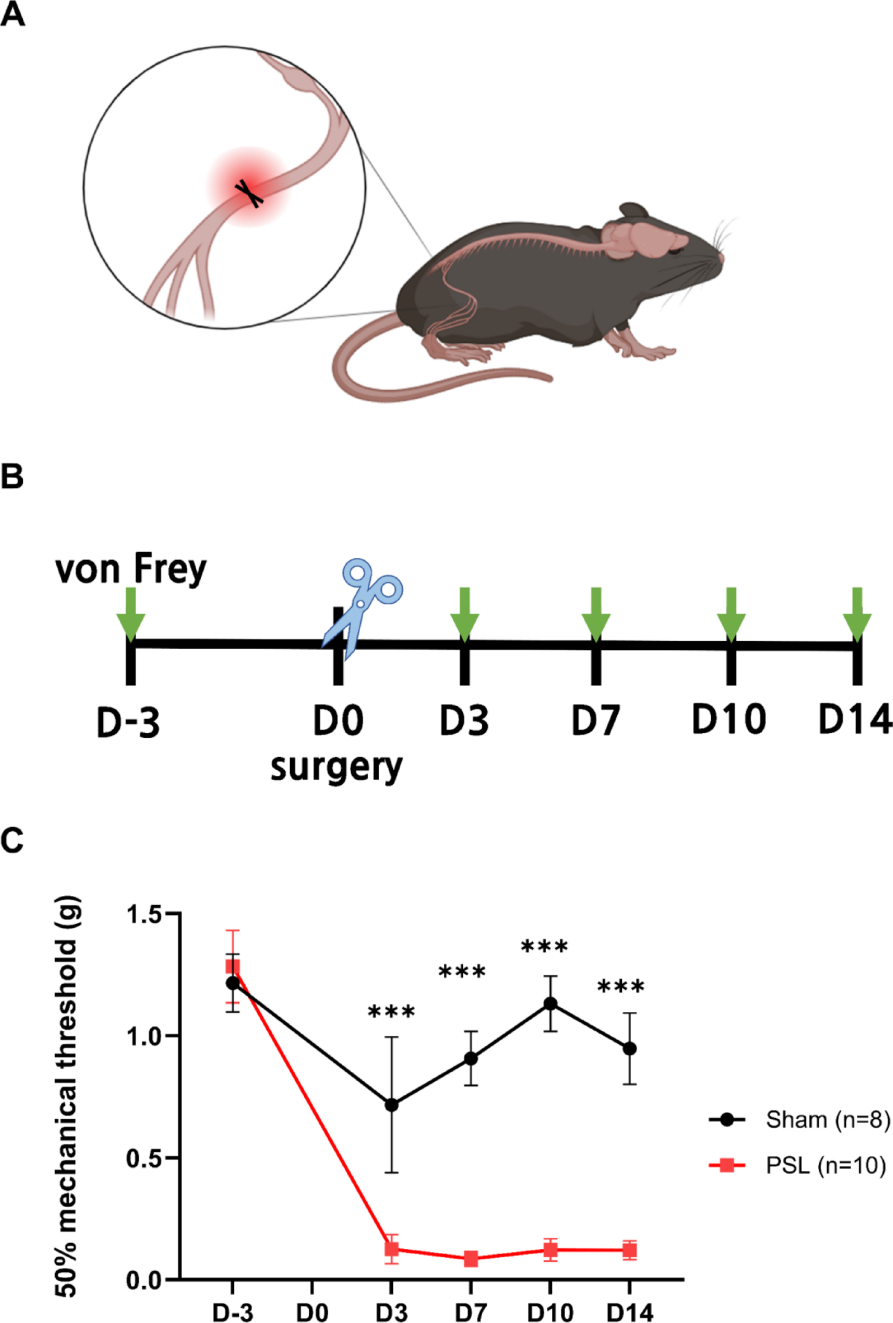
The chronic neuropathic pain induced by partial sciatic nerve ligation in the pain behavioral assessment. **A** Schematic illustration of the establishment of partial sciatic nerve ligation model. **B** An experimental design showing the time-course of allodynia induction and behavioral tests. **C** 50% PWT of the ipsilateral hind paw in mice were evaluated in a time-course study. (Sham, *n* = 8; PSL, *n* = 10). The error bars indicate the SEM. Two-way ANOVA with Tukey’s multiple comparisons test was used. ***p < 0.001

PSL mouse model, an experimental model for studying chronic neuropathic pain [3, 17, 24], was used in this study. PSL surgery was performed on the right sciatic nerve of the animal (Fig. 1A). The mechanical allodynia test was performed utilizing von Frey filament stimuli (Fig. 1B) to calculate 50% paw withdrawal thresholds (PWT). The PWT of the ipsilateral hind paw was significantly decreased 3 days after the surgery and persisted throughout the testing period (Fig. 1C). It demonstrated that mechanical allodynia successfully occurred via PSL surgery.

### Analgesic effects of taVNS on mechanical allodynia

Building on previous findings that taVNS reduces thermal hypersensitivity in a diabetic neuropathy model [25], this study investigated whether taVNS could similarly attenuate mechanical allodynia in a nerve injury-induced neuropathic pain model and sought to determine optimal stimulation parameters for achieving significant analgesic effects.

**Fig. 2:**
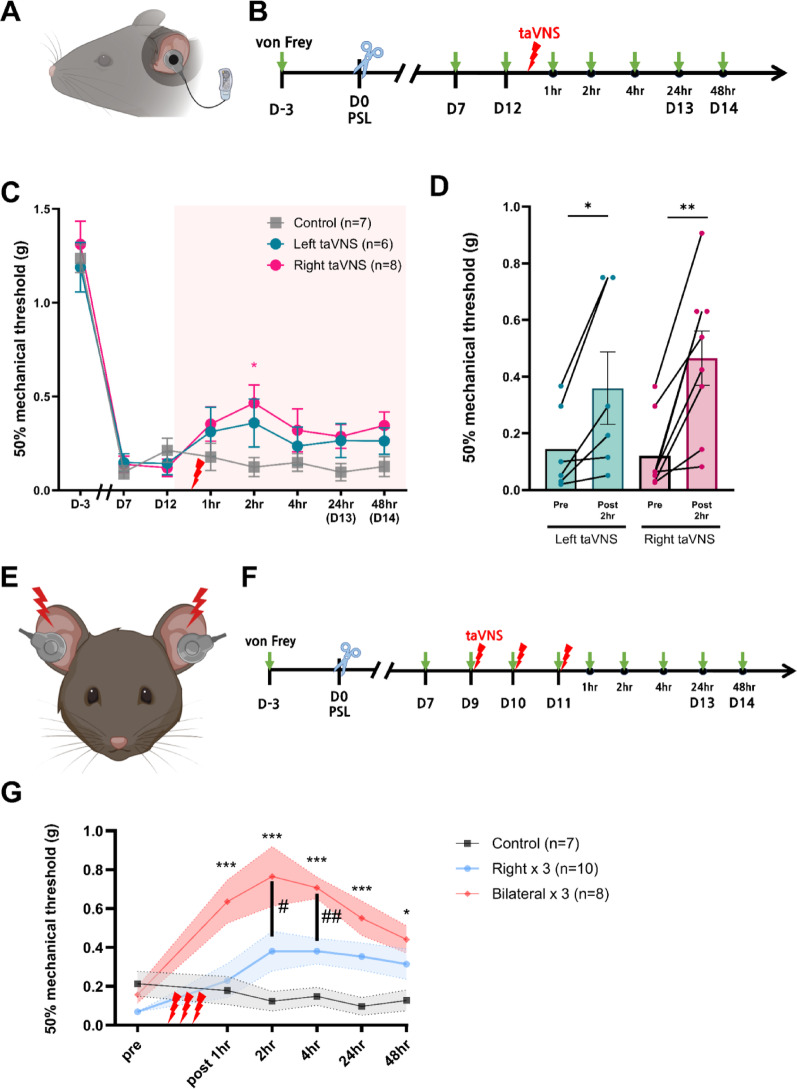
Experimental setup and effects of taVNS on mechanical allodynia in PSL model. **A**,** B** Schematic illustrations of the taVNS stimulation setup targeting ABVN and the experimental timeline. **C** Single-session taVNS treatment revealed a significant reduction in mechanical allodynia in the right-sided stimulation group compared to the control (Control, *n* = 7; Left taVNS, *n* = 6; Right taVNS, *n* = 8). The pink box represents the period during which behavioral responses were observed after taVNS stimulation. Repeated measures two-way ANOVA with Dunnet’s multiple comparisons test was used (Compared to control, 2 h post-stimulation). **D** Both sides showed significant pain relief when compared to pre-stimulation (Left taVNS, *n* = 6; Right taVNS, *n* = 8). **E**,** F** Experimental setup for bilateral taVNS with repeated stimulations and the experimental timeline. **G** Repeated bilateral taVNS sessions resulted in a significantly greater and sustained reduction in mechanical allodynia compared to both the control and unilateral right-sided stimulation groups (Control, *n* = 7; Right taVNS × 3, *n* = 10; Bilateral taVNS × 3, *n* = 8). Error bars indicate SEM. Two-way ANOVA with Tukey’s multiple comparisons test was used. Compared to control group, *** *p* < 0.001; **p < 0.01; *p < 0.05. Compared to unilateral stimulation group, ^##^*p* < 0.01; ^#^*p* < 0.05

In this initial experiment, taVNS was applied to the auricular branch of the vagus nerve (ABVN, Fig. 2A) at 20 Hz, 5 V, square waveform, for 20 min. Stimulation was administered on postoperative day 12 (D12), corresponding to the chronic pain phase, with groups receiving unilateral left, unilateral right, or sham stimulation (anesthesia without stimulation). Mechanical allodynia was assessed using the von Frey filament test at baseline (3 days prior to surgery) and at postoperative days 7 and 12. Day 12 (D12) served as the pre-stimulation baseline, with subsequent assessments conducted at multiple time points post-stimulation (1, 2, 4, 24, and 48 h) to determine the peak analgesic effect. Thus, the 24-h and 48-h measurements corresponded to days 13 and 14 post-surgery (Fig. 2B).

The results showed that the taVNS-treated groups exhibited a significant reduction in mechanical allodynia compared to the sham control group. The unilateral right-sided stimulation (ipsilateral to sciatic nerve injury) displayed a pronounced analgesic effect, peaking at 2 h post-stimulation (Fig. 2C). Unilateral left-sided stimulation also showed significant reduction compared to its pre-stimulation baseline at 2 h (Fig. 2D), although this effect did not reach significance compared to the sham control group (Fig. 2C).

Based on the observed lateral differences and the need for sustained relief, two multi-session conditions were evaluated in subsequent experiments: daily unilateral right-sided stimulation for three consecutive days, and daily bilateral stimulation applied to both sides of the cymba concha over the same three-day period (Fig. 2E, F). These experiments followed a similar protocol, with baseline measurements obtained on days 9, 10, and 11 prior to the first stimulation session. Multi-session bilateral stimulation resulted in a significantly greater reduction in allodynia compared to sham control, with the effect persisting for at least 48 h (Fig. 2G). Furthermore, bilateral stimulation demonstrated significantly superior outcomes compared to daily unilateral right-sided stimulation at the 2-h and 4-h post-stimulation time points (Fig. 2G). These results indicate that daily bilateral taVNS administered over three sessions resulted in the most substantial and prolonged reduction in chronic neuropathic mechanical allodynia, with effects sustained for at least 48 h.

### Verification of c-Fos expression in the DRN and CeA in PSL animals following taVNS application

**Fig. 3:**
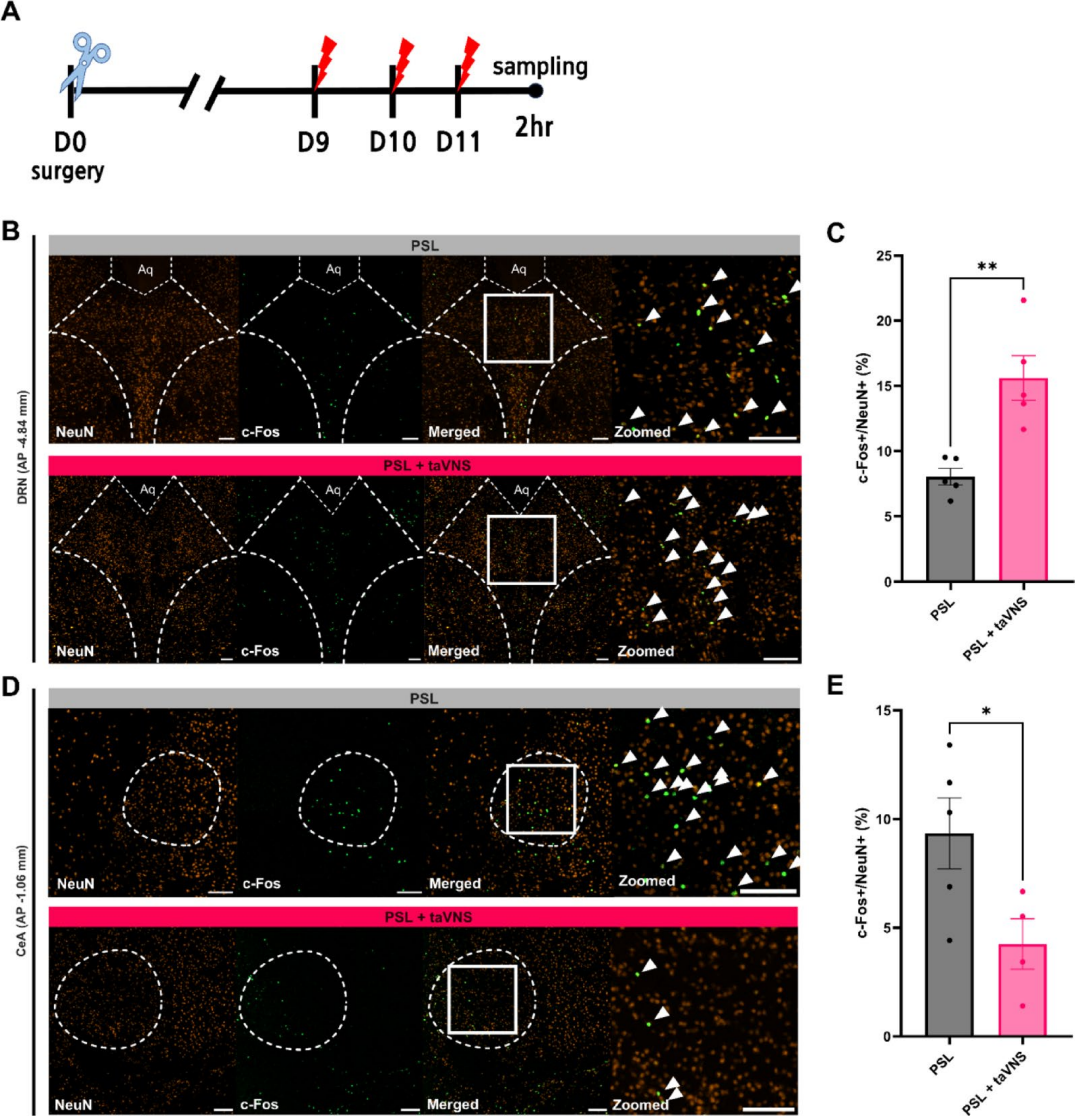
Differential effects of bilateral taVNS on neuronal activation in DRN and CeA. **A** Schematic illustration of the experimental timeline. Bilateral taVNS was applied once daily for three consecutive days (Days 9, 10, and 11) with brain tissue collected 2 h after the final stimulation session. **B** Representative immunofluorescence images showing NeuN (orange) and c-Fos (green) expression in the DRN of the PSL and PSL + taVNS groups. Merged image demonstrates colocalization of activated neurons. Arrowheads indicate NeuN/c-Fos double-positive neurons. Scale bar = 100 μm. **C** Quantification of c-Fos-positive neurons as a percentage of NeuN-positive cells. taVNS significantly increased c-Fos expression in the DRN (*n* = 5 from 4 mice for each group). **D** Representative images of the section including the CeA. Scale bar = 100 μm. **E** Quantification of c-Fos-positive neurons showed taVNS significantly reduced neuronal activation compared to the PSL group in the CeA (*n* = 5 from 4 mice for PSL and *n* = 4 from 4 mice for PSL + taVNS group). Error bars indicate SEM. Unpaired t-test was used for statistical analysis. *p < 0.05, **p < 0.01

Given that VNS and taVNS modify neurochemistry, promote adaptive changes within the brain [26], influences the release of pain modulating neurotransmitters [11, 27], and activate regions like the DRN via the NTS pathway [28] we investigated how taVNS affects neuronal activation in the DRN and the CeA, a key region involved in pain signal integration and affective processing, in the context of the PSL model. Immunohistochemistry was performed on the DRN and CeA using the previously identified optimal stimulation protocol (three daily bilateral sessions). Brain tissue was collected 2 h after the final stimulation session (corresponding to the peak analgesic effect) and double-stained for NeuN (neuronal marker) and c-Fos (neuronal activation marker). The ratio of c-Fos-positive to NeuN-positive cells was quantified for each region (Fig. 3A).

The results revealed a differential response in the DRN and CeA. In the DRN, the taVNS-treated group showed a significantly increased c-Fos/NeuN ratio compared to the PSL model group (Fig. 3B, C). Conversely, the taVNS group exhibited a significantly lower c-Fos/NeuN ratio in the CeA, indicating reduced neuronal activation (Fig. 3D, E). These findings suggest that bilateral taVNS activates the DRN, a major source of serotonin, while concurrently reducing activity in the CeA, a key region in the affective dimension of pain processing. These results underscore a potential mechanism by which taVNS exerts its analgesic effects, involving differential modulation of neuronal activity in the serotonergic pathways and pain circuits.

### Reversal of taVNS-mediated analgesic effect by serotonin depletion in PSL model

**Fig. 4:**
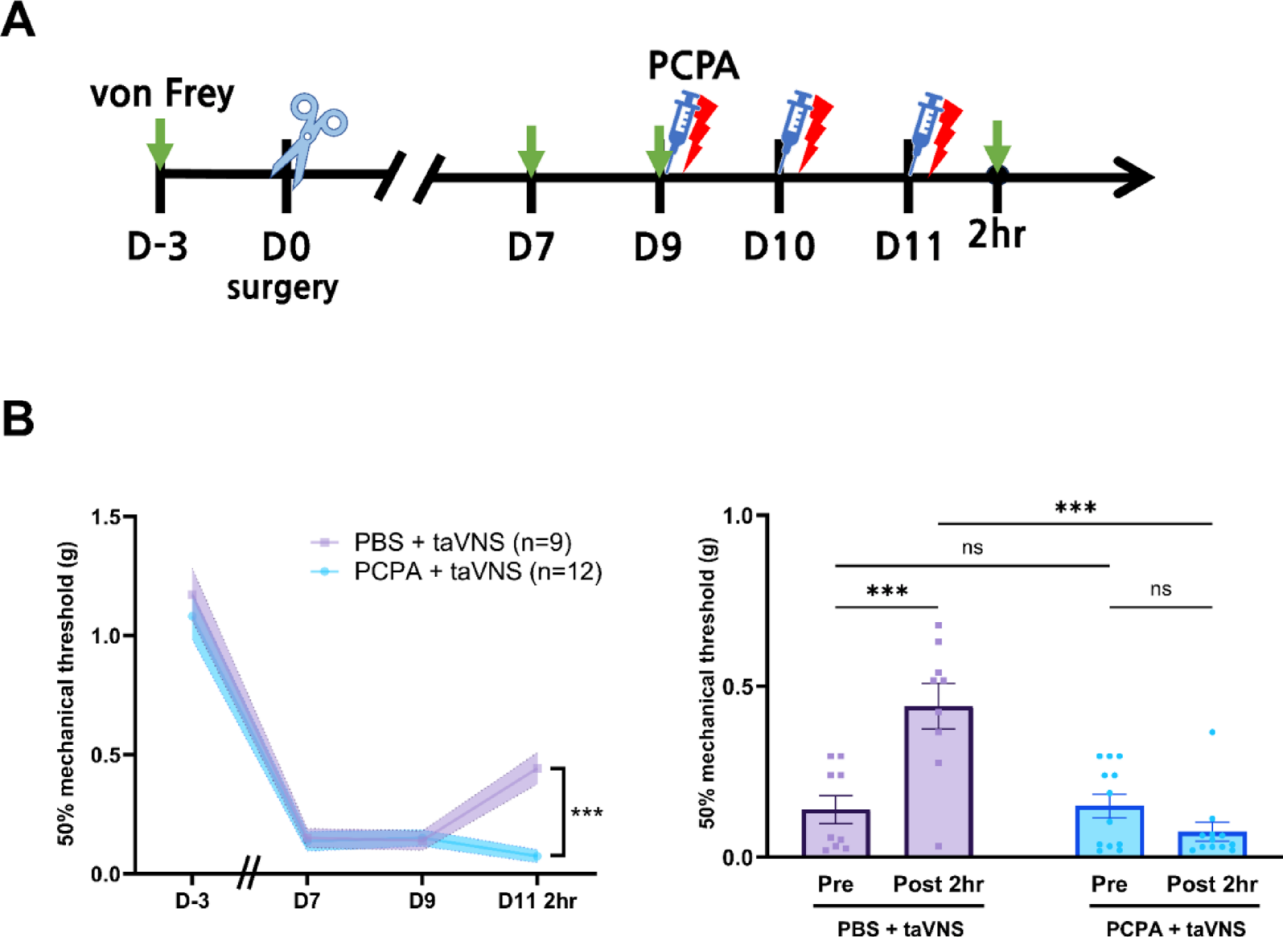
The involvement of serotonin in the analgesic effects of taVNS in PSL model. **A** Schematic timeline of the experimental protocol. Mice received the serotonin synthesis inhibitor PCPA or a vehicle control 30 min prior to each taVNS session on Days 9, 10, and 11. Mechanical thresholds were assessed using the von Frey test before and 2 h after the final taVNS session. **B** Behavioral results comparing taVNS effects with and without serotonin inhibition. The left graph shows no significant difference in mechanical thresholds between the PBS and PCPA groups at baseline (D9), indicating that both groups started with similar pain levels. However, by Day 11, the PBS + taVNS group exhibited a significant improvement in pain threshold compared to the PCPA + taVNS group. The right graph compares pre- and post-stimulation thresholds within each group. The PBS + taVNS group showed a significant increase in pain threshold 2 h post-stimulation, while the PCPA + taVNS group showed no significant change, confirming that serotonin synthesis is essential for taVNS-mediated analgesia. Error bars represent SEM. Two-way ANOVA with Tukey’s multiple comparisons test was used. ***p < 0.001, n.s., not significant

Given the observation that taVNS analgesia was associated with increased c-Fos expression in the serotonergic DRN, we investigated the role of serotonin in mediating these effects. To investigate this, the serotonin synthesis inhibitor PCPA [21] or vehicle was administered to PSL mice 30 min before each taVNS session. Day 9 (D9) served as the pre-PCPA baseline. The taVNS protocol involved three daily bilateral stimulation sessions, and mechanical allodynia was assessed at baseline (Day 9) and 2 h post-stimulation (Fig. 4A). The results demonstrated that PCPA treatment significantly abolished the analgesic effect of taVNS observed in the vehicle-treated group (Fig. 4B). These findings suggest that serotonin synthesis is critical for taVNS to exert its analgesic effects on mechanical allodynia in this neuropathic pain model.

## Discussion

The present study demonstrated that taVNS effectively alleviates mechanical allodynia in a neuropathic pain model induced by PSL. A series of behavioral, immunohistological, and pharmacological experiments were conducted to explore the potential mechanisms underlying the analgesic effects of taVNS. Our findings provide new insights into how taVNS modulates pain processing, particularly through its interaction with the serotonergic system and specific brain regions, such as the DRN and CeA.

### Insights into enhanced analgesic effects of bilateral TaVNS

In this study, repetitive bilateral taVNS demonstrated significantly stronger and longer-lasting analgesic effects compared to repetitive unilateral stimulation as well as single-session stimulation, with these effects persisting for at least 48 h. These findings highlight the importance of stimulation configurations in enhancing the efficacy of taVNS and provide valuable insights for optimizing clinical application. Although the precise mechanisms underlying these differences remain unclear, several possibilities exist.

It is well-established that VNS and taVNS can increase the release of neurotransmitters such as norepinephrine and serotonin, which may enhance analgesic effects by activating descending pain modulation pathways. Bilateral stimulation may engage broader neural networks [29], potentially facilitating simultaneous activation of circuits in both hemispheres. This could modulate connectivity between brain regions involved in pain processing and influence both the sensory and emotional dimensions of pain perception. The therapeutic potential of bilateral taVNS has also been observed for disorders of consciousness, where bilateral stimulation showed a prominent effect to restore consciousness levels. In these studies, bilateral taVNS is believed to promote neurotransmitter release, improving the disrupted brain connectivity. This restoration is thought to facilitate sensory processing and cognitive function, ultimately leading to the recovery of behavioral responses [30].

Further supporting this hypothesis, clinical studies have demonstrated that bilateral taVNS effectively modulates brain regions involved in cognitive and emotional processes. These regions include the insula, posterior temporal lobe, and prefrontal cortex, where significant activation within vagus nerve-associated networks has been observed [31]. This activation is attributed to the interaction between taVNS signaling and broader neural architecture, potentially enhancing neural plasticity and connectivity.

Taking these findings into account, bilateral taVNS likely enhances neurotransmitter release and improves neural network integration, contributing to more effective pain modulation and the recovery of related neural functions. These findings underscore the necessity for further research to optimize stimulation parameters and elucidate the mechanisms underlying the broader therapeutic effects of bilateral taVNS.

### Exploring the roles of DRN and CeA in taVNS-induced pain modulation

This study investigated the analgesic effects and underlying mechanisms of taVNS in a neuropathic pain model induced by PSL. The application of taVNS resulted in changes in neuronal activity in the DRN and CeA. Furthermore, the administration of PCPA, a serotonin synthesis inhibitor, abolished the analgesic effects of taVNS, indicating that serotonin is closely associated with its pain-relieving effects. However, further research is required to explore the specific roles of the DRN and CeA and their causal interactions in taVNS-induced analgesia.

To elucidate the mechanisms of taVNS, neuronal activity changes across various brain regions were analyzed using c-Fos expression as a marker. Following taVNS treatment, an increase in c-Fos expression was observed in the DRN, which may indicate increased serotonin release due to DRN activation [16, 32]. This enhanced serotonin release likely contributed to pain modulation and the promotion of neuroplasticity [33]. Previous studies have also reported that taVNS increases serotonin release, which improves neuroplasticity and facilitates nerve regeneration [34–39].

The CeA, primarily composed of GABAergic neurons, is a key region involved in emotional regulation and is associated with anxiety and fear responses. Reduced neuronal activity in the CeA is directly linked to decreased pain perception [40], consistent with the reduced c-Fos expression observed in this study. Similarly, fMRI studies have demonstrated that taVNS decreases activation in limbic regions such as the CeA and hippocampus, highlighting its role in emotional and pain regulation [41]. Moreover, taVNS has been shown to influence emotion-regulating regions, including the CeA, through its electrical signals [42]. Clinical evidence also suggests that taVNS improves symptoms of depression and emotional dysregulation [43–45], further supporting its role in modulating emotional states, which are closely linked to chronic pain.

The serotonergic pathway between the DRN and CeA provides a potential explanation for these findings. Serotonin released from the DRN inhibits the activity of somatostatin-positive (SOM+) neurons in the CeA via 5-HT1A receptors, thereby regulating emotional and pain processing [46]. However, in chronic neuropathic pain, DRN activity can decrease, leading to reduced serotonin levels in the CeA and subsequent overactivation of SOM + neurons, which exacerbates pain and emotional dysregulation [47]. Our results suggest that taVNS may counteract this by increasing DRN activity and serotonin release, thus inhibiting the overactive CeA and alleviating both sensory and affective components of neuropathic pain.

In conclusion, taVNS alleviates neuropathic pain via serotonin-dependent pathways, and the analgesic effects was enhanced with bilateral and repetitive stimulation. Along with the analgesic effects of taVNS, increased DRN activity and decreased CeA activity were observed. These findings suggest that serotonin release through DRN activation contributes to pain suppression, while suppression of excessive neuronal activity in the CeA is related to pain inhibition. These findings highlight the potential of taVNS as a non-invasive therapeutic approach for managing neuropathic pain.

## Data Availability

No datasets were generated or analysed during the current study.
